# Deep learning and alignment of spatially resolved single-cell transcriptomes with Tangram

**DOI:** 10.1038/s41592-021-01264-7

**Published:** 2021-10-28

**Authors:** Tommaso Biancalani, Gabriele Scalia, Lorenzo Buffoni, Raghav Avasthi, Ziqing Lu, Aman Sanger, Neriman Tokcan, Charles R. Vanderburg, Åsa Segerstolpe, Meng Zhang, Inbal Avraham-Davidi, Sanja Vickovic, Mor Nitzan, Sai Ma, Ayshwarya Subramanian, Michal Lipinski, Jason Buenrostro, Nik Bear Brown, Duccio Fanelli, Xiaowei Zhuang, Evan Z. Macosko, Aviv Regev

**Affiliations:** 1 Broad Institute of MIT and Harvard, Cambridge, MA USA; 2 Department of Physics and Astrophysics, University of Florence, Florence, Italy; 3 Northeastern University, Boston, MA USA; 4 Department of Chemistry and Chemical Biology, Department of Physics, Harvard University, Cambridge, MA, USA; 5 School of Engineering and Applied Sciences, Harvard University, Cambridge, MA, USA; 6 Department of Biology, MIT, Cambridge, MA USA; 7 Department of Stem Cell and Regenerative Biology, Harvard University, Cambridge, MA USA; 8 Present Address: Genentech, South San Francisco, CA USA; 9 Present Address: Roche, Monza, Italy; 10 Present Address: School of Computer Science and Engineering, Racah Institute of Physics, Faculty of Medicine, The Hebrew University, Jerusalem, Israel; 11 Present Address: Howard Hughes Medical Institute, Chevy Chase, MD, USA

**Keywords:** Machine learning, Neuroscience, Software, Transcriptomics, Imaging

## Abstract

Charting an organs’ biological atlas requires us to spatially resolve the entire single-cell transcriptome, and to relate such cellular features to the anatomical scale. Single-cell and single-nucleus RNA-seq (sc/snRNA-seq) can profile cells comprehensively, but lose spatial information. Spatial transcriptomics allows for spatial measurements, but at lower resolution and with limited sensitivity. Targeted in situ technologies solve both issues, but are limited in gene throughput. To overcome these limitations we present Tangram, a method that aligns sc/snRNA-seq data to various forms of spatial data collected from the same region, including MERFISH, STARmap, smFISH, Spatial Transcriptomics (Visium) and histological images. Tangram can map any type of sc/snRNA-seq data, including multimodal data such as those from SHARE-seq, which we used to reveal spatial patterns of chromatin accessibility. We demonstrate Tangram on healthy mouse brain tissue, by reconstructing a genome-wide anatomically integrated spatial map at single-cell resolution of the visual and somatomotor areas.

## Abstract (web summary)

Tangram is a versatile tool for aligning single-cell and single-nucleus RNA-seq data to spatially resolved transcriptomics data using deep learning.

## Main

A Human Cell Atlas^1–3^ should combine high-resolution molecular and histological mapping with anatomical and functional data. Advances in single-cell and spatial genomics^4^ opened the way to high-resolution spatial profiles, but each of the currently available technologies addresses only some of the challenge of resolving entire transcriptomes in space at single-cell resolution. On the one hand, sc/snRNA-seq profiles single cells transcriptome-wide, from which we can recover cell types^5^, gene expression programs^6,7^, and developmental relations^8,9^, but by necessity lose direct spatial information. Conversely, spatial technologies resolve transcriptomes in space, but are limited in either gene throughput or spatial resolution. In general, targeted in situ technologies (such as in situ sequencing^10^, multiplexed error-robust fluorescence in situ hybridization (MERFISH)^11^, single-molecule FISH (smFISH)^12^, cyclic-ouroboros smFISH (osmFISH)^13^, spatially resolved transcript amplicon readout mapping (STARmap)^14^, targeted expansion sequencing^15^, and sequential FISH (seqFISH+)^16^) are typically limited to hundreds of preselected genes, but adding more probes can reduce accuracy for some genes^14^. Spatial transcriptomics methods (such as Spatial Transcriptomics (ST/Visium)^17^, Slide-seq^18^, and High Definition Spatial Trascriptomics^19^) spatially barcode entire transcriptomes, but with limited capture rate (and substantial ‘dropouts’, which increase at higher resolution^19^) and a spatial resolution larger than a single cell, ranging from 50 µm to 100 μm for ST to 10 μm for Slide-seq. In addition, for biological interpretation, cellular features would ideally be related to the histological or organ scale, which is conventionally done using methods from computer vision for registration of medical images^20,21^. However, these methods typically require human supervision, such as identification of anatomical landmarks in images, preventing the complete automation that is desirable for organ-scale mapping.

Computational methods have previously bridged this gap by combining single-cell and spatial measurements^22–25^. These methods can reconstruct key landmark genes by leveraging local alignment in transcriptome space^22–24^, or hypotheses such as continuity in gene expression^25^. However, intrinsically sparse or granularly distributed genes are difficult to predict. For measurements at coarse spatial resolution, computational methods aim to deconvolve these data^18,26^, by either learning a program dictionary^18^ or a probability distribution of the data^26^, to infer a cell-type composition within a spatial voxel. However, deconvolution is hindered by spatial ‘dropouts,’ in which cell types defined by sparse or dim markers are not correctly detected^27^.

Here, we present Tangram, a deep-learning framework to address two challenges: learn spatial gene-expression maps transcriptome-wide at single-cell resolution, and relate those to histological and anatomical information from the same specimens. Tangram learns a spatial alignment of sc/snRNA-seq data from a reference spatial data of any kind—either fine or coarse grained—as we demonstrate by spatially mapping snRNA-seq data from the isocortex of the adult healthy mouse brain using each of five kinds of spatial supports, at different levels of resolution and gene coverage: ISH, smFISH, Visium (Spatial Transcriptomics), STARmap and MERFISH. Tangram produces consistent spatial maps of cell types and overcomes limitations in throughput or resolution. It corrects low-quality genes, even in high-resolution methods, provides single-cell resolution for low-resolution methods, and provides genome-wide coverage for targeted methods. By mapping multimodal single data (simultaneous high-throughput ATAC and RNA expression with sequencing (SHARE-seq)^28^) on spatial support, Tangram visualizes spatial patterns of chromatin accessibility and transcription factor motif scores at single-cell resolution. Finally, Tangram includes a dedicated new computer vision module that leverages histological data, and maps it to anatomical positions in an existing Common Coordinate Framework in the brain. If a histology image is available, even without any further annotation, this module relates all scales, to a single integrated atlas.

## Results

### Tangram: learning of spatially resolved single-cell transcriptomes by alignment

**Fig. 1:**
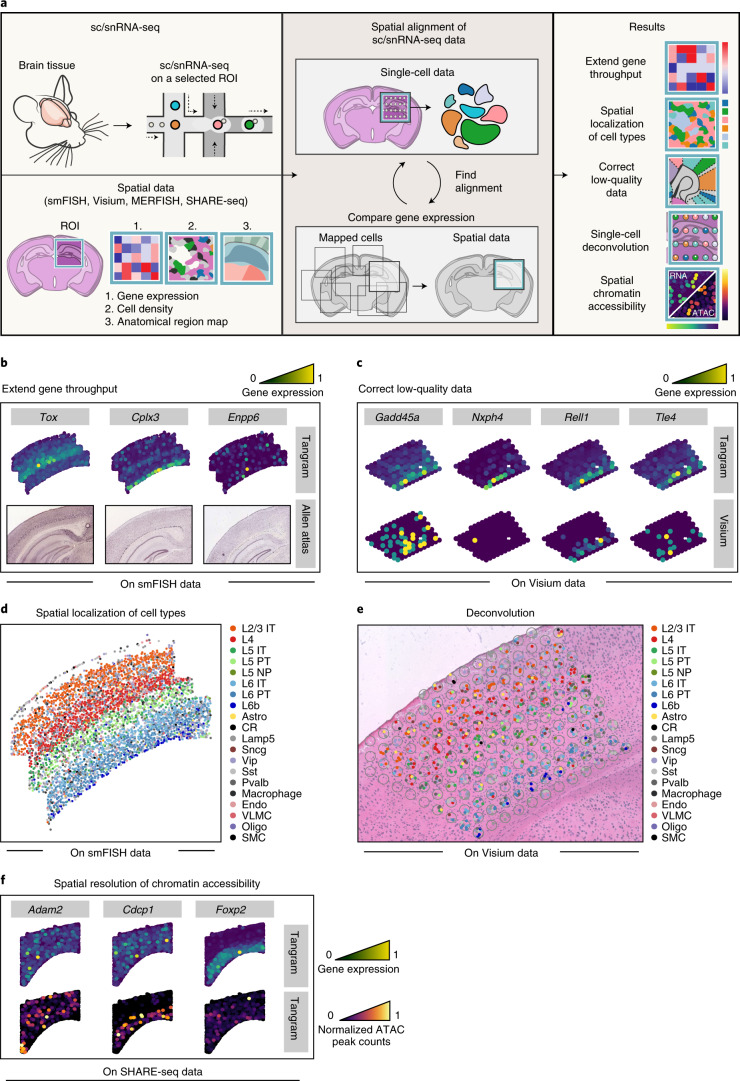
Tangram learns spatial transcriptome-wide patterns at single-cell resolution from sc/snRNA-seq data and corresponding spatial data. **a**, Overview. sc/snRNA-seq data and spatial data, collected from the same tissue, are spatially aligned by comparing gene expression of their shared genes. **b**–**f**, Tangram use cases. **b**, Generating genome-wide spatial patterns from gene signature data. Predicted expression patterns (color bar, normalized mRNA counts, see Methods) for each of three genes not included in an input smFISH dataset are validated against their corresponding images from the Allen ISH atlas (bottom). **c**, Correction of low-quality data for spatially measured genes. Predicted (top) and measured (bottom, by Visium) expression patterns (color bar, normalized mRNA counts, see Methods) of four known markers, the correct localization of which is missing in direct Visium measurements but recovered in the predicted patterns. **d**, Cell-type localization. Spatial distribution of cell types defined by snRNA-seq (legend) mapped on a smFISH brain slide. **e**, Single-cell deconvolution of lower-resolution Spatial Transcriptomics. Predicted single cells (colored dots, legend) in each Visium voxel (gray circle) based on snRNA-seq data mapped onto a Visium slide. **f**, Spatially resolved chromatin patterns. Predicted spatial gene expression (top, color bar, normalized mRNA counts, see Methods) and chromatin accessibility (bottom; color bar, normalized ATAC peak counts, see Methods) by mapping the RNA component of SHARE-seq data to a MERFISH slide.

**Fig. 2:**
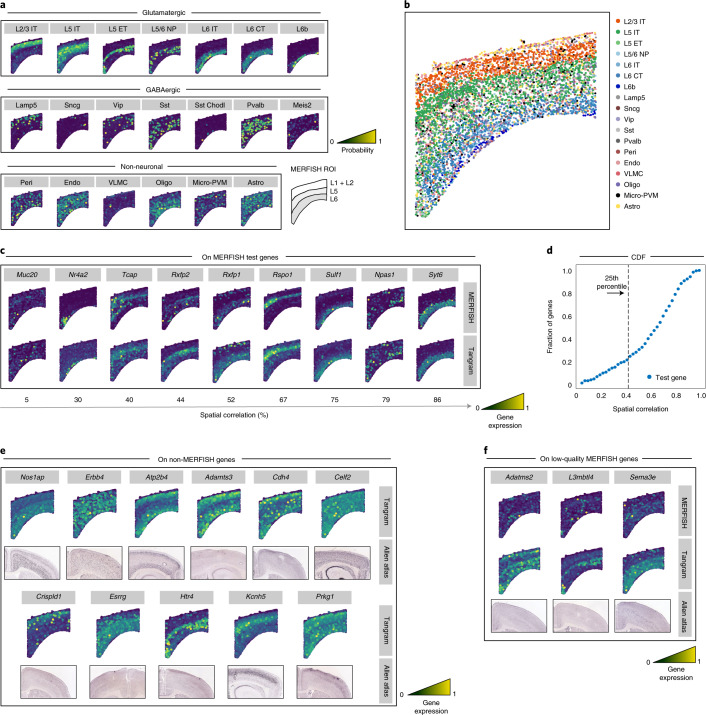
Tangram maps cells with high-resolution MERFISH measurements and expands them to genome scale. **a**, Probabilistic mapping of snRNA-seq data on MERFISH data. Probability of mapping (color bar) of each cell subset (gray label) in each of three major categories. Bottom right, schematic of key layers. **b**, Deterministic mapping. MERFISH slide with segmented cells (dot) colored by the cell-type annotation of the most likely snRNA-seq profile mapped on that position by Tangram (legend). **c**,**d**, Predicted expression of test genes. **c**, Measured (top) and Tangram-predicted (bottom) expression (color bar signifies fluorescence at top and normalized mRNA counts at bottom, see Methods) of select test gene (gray labels) with different extents of spatial correlation (bottom arrow, %) between measured and predicted patterns. **d**, Cumulative distribution function (CDF) of spatial correlation (*x* axis) between predicted and measured patterns for test genes. Dashed line: 75% of test genes are predicted with spatial correlation >40%. **e**, Predicted expression of test genes. Tangram-predicted (bottom) expression (top; color bar, normalized mRNA counts, see Methods) and corresponding ISH images from the Allen Brain Atlas (bottom) for 11 genes not measured by MERFISH. **f**, Correction of low-quality spatial measurements. MERIFSH measured (top), Tangram-predicted (middle) and Allen Brain Atlas ISH, for genes where predicted patterns differ from MERFISH measurement but match direct inspection of Allen ISH images (color bar, normalized mRNA counts, see Methods).

**Fig. 3:**
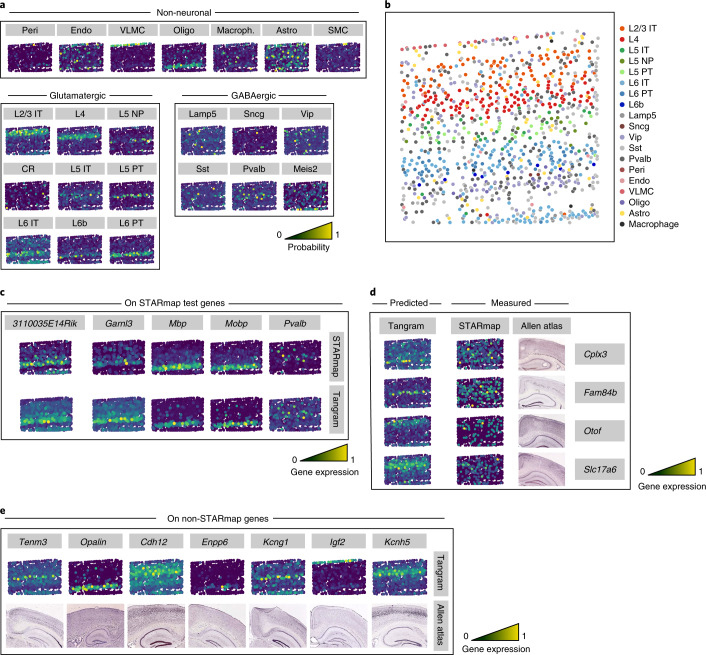
Correction of low-quality genes by mapping snRNA-seq on STARmap data. **a**, Probabilistic mapping of snRNA-seq data on STARmap data. Probability of mapping (color bar) of each cell subset (gray label) in each of three major categories. **b**, Deterministic mapping. STARmap slide with segmented cells (dot) colored by the cell-type annotation of the most likely snRNA-seq profile mapped on that position by Tangram (legend). **c**, Measured (top) and Tangram-predicted (bottom) expression (color bar signifies fluorescence at top and normalized mRNA counts at bottom, see Methods) of select test gene (gray labels). **d**, Correction of low-quality spatial measurements. Tangram-predicted test genes (left), STARmap measurements (middle), and Allen atlas images (right) (color bar, normalized mRNA counts, see Methods) of four genes (gray labels) whose predicted patterns differ from STARmap measurement but match direct measurement by MERFISH. **e**, Predicted expression of test genes. Tangram-predicted (top) expression (top; color bar, normalized mRNA counts, see Methods) and corresponding ISH images from the Allen Brain Atlas (bottom) for six genes not measured by STARmap.

**Fig. 4:**
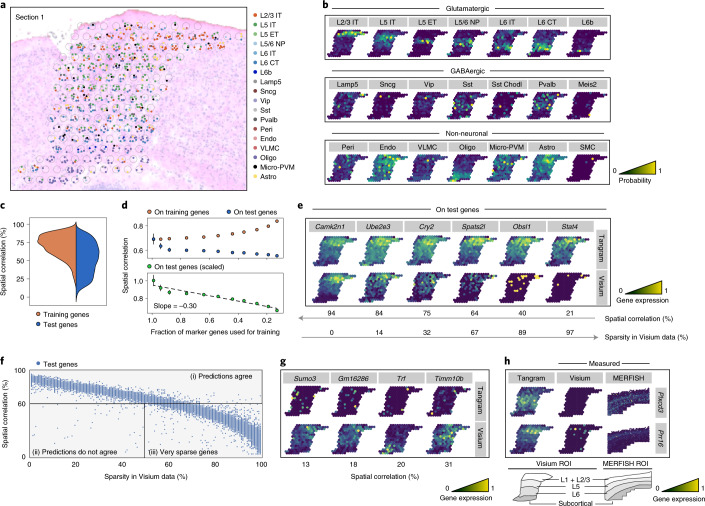
Mapping snRNA-seq data to Spatial Transcriptomics data (Visium) demonstrates deconvolution and imputation of dropouts. **a**, Single-cell deconvolution. Predicted single cells (colored dots, legend) in each Visium voxel (gray circle) based on snRNA-seq data mapped onto a Visium slide. Cell assignment within a voxel is random with respect to the specific segmented cell. **b**, Probabilistic mapping of snRNA-seq data on the Visium ROI. Probability of mapping (color bar) of each cell subset (gray label) in each of three major categories. **c,d**, Predicted expression of test and training genes. **c**, Normalized (that is, unit area) distribution of single-gene spatial correlation coefficients (*y* axis) between Tangram-predicted and Visium-measured patterns in training (orange) and test (blue) genes. **d**, Reducing the number of training genes decreases prediction performance. Spatial correlation (*y* axis, top) for training genes (orange) and test genes (blue), and scaled spatial correlation (*y* axis, bottom) for test genes (scaled by the correlation averaged across training genes) for Tangram models learned with different fractions of 1,237 input training genes (*x* axis). **e**–**h**, Impact of Visium data sparsity on prediction and correction. **e**, Tangram-predicted (top) and Visium-measured (bottom) expression (color bar, normalized mRNA counts, see Methods) of six select test genes (gray labels) with different extents of spatial correlation between measured and predicted patterns (top arrow, %) and of Visium data sparsity (bottom arrow, %). **f**, Spatial correlation of test genes is negatively correlated to sparsity in Visium data. Spatial correlation (*y* axis) between measured and predicted patterns for test genes (blue dots) and their corresponding measurement sparsity (*x* axis). Lines delineate three regions according to model performance. **g**, Few low-sparsity genes are not predicted well. Tangram-predicted (top) and Visium-measured (bottom) expression (color bar, normalized mRNA counts, see Methods) of four genes (gray labels) with low sparsity that are not well-predicted by model (from region (ii) of **f**). **h**, Correction of low-quality spatial measurements. Tangram-predicted (left), Visium (middle) and MERFISH (right) measurements (color bar signifies fluorescence for MERFISH figure, normalized mRNA counts for all others, see Methods), of two genes (gray labels) whose predicted patterns differ from Visium measurements but match direct measurement by MERFISH, and are highly sparse in Visium measurements (from region (iii) of **f**).

We developed Tangram, an algorithm that uses sc/snRNA-seq data as ‘puzzle pieces’ to align in space to match ‘the shape’ of the spatial data (Fig. 1a). The input to Tangram is sc/snRNA-seq data along with spatial profiling data from the same region or tissue type, from any currently available spatial method (for example MERFISH, smFISH, STARmap, ISH, or Visium), requiring only that the two modalities share at least some subset of common genes. Intuitively, Tangram first randomly places the sc/snRNA-seq profiles in space, then computes an objective function that mimics the spatial correlation between each gene in the sc/snRNA-seq data and in the spatial data. Tangram then rearranges the sc/snRNA-seq profiles in space to maximize the total spatial correlation across the genes shared by the datasets. When Tangram finishes, the mapped sc/snRNA-seq profiles constitute the new spatial data: they now contain all genes at single-cell resolution and with spatial position. From the learned mapping function, Tangram can (1) expand from a measured subset of genes to genome-wide profiles (Fig. 1b); (2) correct low-quality spatial measurements (Fig. 1c); (3) map the location of cells of different types (Fig. 1d); (4) deconvolve low-resolution measurements to single cells (Fig. 1e); and (5) resolve spatial patterns of chromatin accessibility at single-cell resolution by aligning multimodal data (Fig. 1e).

Technically, Tangram is based on nonconvex optimization (Methods), in which the Tangram optimization function rewards the spatial alignment of sc/snRNA-seq data using two similarity functions: cell-density distributions are compared using Kullback–Leibler (KL) divergence, whereas gene expression is assessed through cosine similarity. If the sc/snRNA-seq data contain more cells than the spatial data (which is the typical case), a filter term in the loss function ensures that only the optimal subset of sc/snRNA-seq observations is selected. The output is a probabilistic mapping, namely, a matrix denoting the probability of finding each cell from the sc/snRNA-seq data in each voxel of the spatial data. From this matrix, we can obtain a deterministic mapping by assigning the most likely sc/snRNA-seq cell to each spatial voxel. Tangram does not contain any hyperparameters, maps a hundred thousand cells in a few minutes (using a single P100 GPU), and is released as PyTorch module.

### Tangram maps cells with MERFISH measurements to generate genome-scale high-resolution expression maps

To apply Tangram, we collected 160,000 snRNA-seq profiles using droplet-based RNA-seq (10Xv3, see for example ref. ^29^), as part of the BRAIN Initiative Cell Census Network (BICCN), from the primary motor area (MOp) of healthy adult mouse brain. Each profile contains the expression of about 27,000 genes, and was annotated following the recently delineated cell-type taxonomy of neocortical areas^30^, to 22 subsets (hereafter, ‘cell types’)^31^. We first mapped these snRNA-seq data with a high-resolution MERFISH dataset of 254 genes, on a section segmented to 4,234 cells (Fig. 2). We trained Tangram using 253 MERFISH genes (all genes but one were detected in our snRNA-seq data). Fifty percent of the aligned profiles were neuronal, with a 6:1 ratio between glutamatergic and GABAergic cells, in accordance with their ratios in snRNA-seq.

To reveal the spatial distribution of cell types, we combined the learned probabilistic mapping with the cell-type annotations in the snRNA-seq data, and obtained a spatial probability distribution for each cell type (Fig. 2a). Glutamatergic cells showed distinct cortical layer patterns of neuronal subpopulations, whereas most, but not all, non-neuronal cells and GABAergic neurons are granularly distributed, as expected. Exceptions included non-neuronal VLMC cells (strongly localized in the first layer) and GABAergic Vip and Lamp5 cells, which appeared to be more concentrated toward the upper layers. To verify that these distributions were not an artifact of our probabilistic approach, we also visualized the cell-type assignment from the deterministic mapping (that is, only the most likely cell is assigned to each spatial location) and observed similar patterns (Fig. 2b).

The learned Tangram model predicted spatial expression patterns well, as demonstrated by a leave-one-out analysis (Methods). As an evaluation score, we computed the spatial correlation between each gene’s real measurement and the predicted spatial pattern of that gene by the learned model. Overall, 75% of the 253 MERFISH genes are predicted with a correlation of >40% (Fig. 2d). To interpret these spatial correlations, we chose nine genes with varied scores and visually compared the predicted spatial patterns with the MERFISH measurements (Fig. 2c). Importantly, the spatial patterns had good qualitative agreement for a broad range of spatial correlation values. For example, the prediction for *Tcap* (40% correlation) is in good accordance with its measurement. This is because when spatial resolution is at the single-cell level, correlation is highly sensitive to noise in gene expression or its measurement, such that a somewhat lower correlation does not imply qualitative disagreement. This phenomenon is especially evident in very sparse genes (such as *Muc20*): the sparse pattern is recapitulated, but the specific single-cell locations are not precise, which may reflect the true nature of these patterns.

Mapping snRNA-seq data on MERFISH increases gene throughput to 27,000 genes, which we validated for 11 selected genes with available ISH data in the Allen ISH dataset (Fig. 2e). Some genes exhibit strong, localized, patterns in striking similarity to those in the Allen images (*Kcnh5*, *Nos1ap*, *Erbb4*, *Atp2b4*, *Celf2*, *Crispld1*). For other genes, the signal in the Allen ISH image is very dim compared with our predictions (*Esrrg*, *Cdh4*, *Adamts3*, *Htr4*, *Prkg1*), but a close inspection reveals agreement as well. This suggests that Tangram can reveal spatial patterns for genes with low expression, as we will further demonstrate below (with Visium data). Notably, we obtained similar results when we predicted withheld genes that were measured by MERFISH but had relatively lower quality, possibly because of less optimal oligonucleotide probes used for these genes: the model predictions were consistent with ISH data, suggesting that the model can ‘correct’ lower quality signal (Fig. 2f).

### Accurate correction of transcripts measured with STARmap

To further investigate Tangram’s correction of low-quality in situ transcripts, we analyzed a STARmap dataset^14^, in which 1,020 genes are measured in 972 cells in a mouse brain slice from the visual area (VISp). We mapped 11,759 SMART-Seq2 (ref. ^30^) snRNA-seq profiles from the VISp area using 995 training genes present in both STARmap and snRNA-seq data.

Inspecting cell-type distributions from either probabilistic (Fig. 3a) or deterministic (Fig. 3b) mapping (Methods), we confirmed that cell-type patterns are consistent with those obtained with MERFISH from the motor area (Fig. 2a,b). Despite a minor cell-type annotation difference between the VISp and MOp snRNA-seq datasets, our model provides robust mapping. For example, while only the VISp (but not MOp) snRNA-seq dataset has an annotated glutamatergic L4 (layer four) cell subset, the model successfully revealed L4 in the MOp data (Fig. 3a) from predicting its marker genes (for example, *Kcnh5* in Figs. 2e and 3e). Finally, the STARmap dataset also contains subcortical tissue (defined as cells below the L6b layer), which allows us to further validate predictions by observing an expected subcortical concentration of oligodendrocytes (Fig. 3a).

Remarkably, Tangram not only predicted expression for genes that were not measured by STARmap, but effectively corrected the spatial expression of low-quality genes (Fig. 3c–e), as compared with the performance of Allen Brain Atlas (http://atlas.brain-map.org/atlas?atlas=1) ISH. First, when holding out each individual STARmap gene from the training, the predicted expression was typically consistent with direct measurements (Fig. 3c). Interestingly, for some genes, our predicted localized patterns were not observed in measurements, especially for lower quality genes (Fig. 3d). Remarkably, in these cases, the predicted pattern agreed well with images from the Allen Brain ISH Atlas (Fig. 3d), confirming the accuracy of our predictions, and Tangram’s ability to correct gene expression of low-quality data. Finally, Tangram correctly predicted the expression of genes that were not measured by STARmap, including markers of cortical layers (*Tenm3*, *Cdh12*, *Kcng1*, *Igf2*) or subcortical tissue (*Opalin* and *Enpp6*), as assessed by their consistency with the Allen Brain ISH Atlas (Fig. 3e).

### Single-cell deconvolution and histological data incorporation with Spatial Transcriptomics

Next, we focused on the deconvolution challenge in the context of lower resolution Spatial Transcriptomics (Visium) data measuring 31,053 genes within 50-μm-diameter circular spots in 3 mouse coronal brain slices (Fig. 4). This was followed by an H&E stain of the slice (section 1), spanning about 160 circular spots on a region of interest (ROI). Single cells are visible in the stained images, so we segmented cells (Methods) to directly estimate cell number within each spot, and counted 939 cells overall.

**Extended Data Fig. 1:**
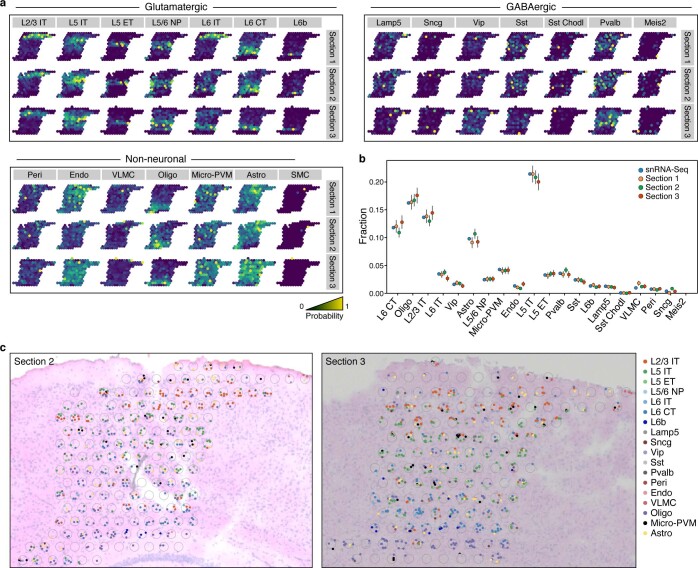
Mapping results on Visium data are consistent across three datasets. **a**. Consistent probabilistic maps across models trained from replicate datasets. Probability of mapping (color bar) of each cell subset (gray label) from each of 3 major categories in models trained separately from three Visium sections (rows). *Section I* is the same shown in Fig. 3b. **b,c**. Consistent deconvolution across models trained from replicate datasets. **b**. Fraction of cells (*y* axis) of each cell type (*x* axis) obtained after deconvolution with models trained separately by each of three Visium sections and in snRNA-seq. **c**. Predicted single cells (colored dots, legend) in each Visium voxel (grey circle) based on snRNA-seq data mapped onto Visium section 2 (left) and section 3 (right) (compare to Fig. 3b). Cell assignment within a voxel is random with respect to the specific segmented cell.

For deconvolution, we first assigned a discrete number of cells to each voxel (matching the number of segmented cells) and then performed a deterministic mapping of each of the cells within each voxel (Methods) to obtain a cell-type localization prediction at single-cell resolution (Fig. 4a). We trained Tangram with a subset of the >30,000 genes by selecting 1,237 training genes as the union of the top 100 marker genes of each cell type in the primary motor cortex (MOp) snRNA-seq data (using a standard pipeline, Methods) that were detected in the Visium profiles. We found that mapped cell-type ratios and those from the snRNA-seq data were consistent (Extended Data Fig. 1b). Our mappings were also robust, as demonstrated by analysis of two other Visium datasets: a coronal section (section 2) consecutive to section 1, and a coronal section collected at approximately the same posterior position, which is publicly available (section 3) (https://support.10xgenomics.com/spatial-gene-expression/datasets/1.1.0/V1_Adult_Mouse_Brain?) (Extended Data Fig. 1c). Assignment within a voxel is random: the model may predict that one microglia cell is contained in a certain voxel, but not which cell it is.

### Tangram imputation of dropouts in Spatial Transcriptomics

Next, we probabilistically mapped the MOp snRNA-seq profiles corresponding to the dissected region for all three Visium slices (Methods). Tangram’s mapping yielded higher resolution, finely localized, cell types (Fig. 4b, Extended Data Fig. 1a). This included correct localization of L6b^+^ glutamatergic neurons, a more concentrated presence of Vip^+^ and Lamp5^+^ GABAergic neurons in upper layers, and positioning of Sst^+^ and Pvalb^+^ GABAergic neurons in deeper layers and of Meis2^+^ and Sst^+^Chodl^+^ GABAergic neurons in rare sparse cell types. In a few cases, there was variation in the mapping between independent experiments, which is consistent with biological variation. For example, colocalization of cell types (for example Sncg^+^ and Vip^+^ GABAergic neurons) is detected across slices from the same batch (section 1 and section 2) but not in section 3; L6 IT cells are more localized in layer 6 in slice 3; and Vip^+^ neurons are more uniformly distributed in section 3 than in section 1 and section 2. These findings are consistent with our expectations.

Notably, Tangram correctly predicted spatial expression patterns from the mapped cells, when we withheld those genes in the training and then compared them with the Visium measurements (Fig. 4c–f). Specifically, we partitioned the genes into 1,237 training genes and 29,816 test genes unseen in the learning of the model, and used spatial correlation as before (Fig. 4c). The 90th quantile of spatial correlation coefficients of training genes is >62%, and 50% of the test genes exceeded this threshold (Fig. 4c,d). As the number of training genes was reduced from 1,237 to 123, so did the relative prediction accuracy (Fig. 4d), although it remained substantial. Inspection of spatial patterns from select test genes showed that, although our predictions always result in a localized pattern in the upper layer, agreement against Visium measurements deteriorates as the gene is more sparsely detected in the original Visium experiment (Fig. 4e, where sparsity is defined as the fraction of voxels in which the gene is undetected).

We hypothesized that this poorer agreement could be due to technical ‘dropouts’’ (~15,000 test genes are entirely undetected in our Visium datasets). Supporting this hypothesis, there is a strong correlation between our prediction scores and data sparsity (Fig. 4f): 98% of nonsparse genes (sparsity < 50%) are correctly predicted by our model (spatial correlation >62% threshold; Fig. 4f, region i); only about 70 nonsparse genes were are not well predicted (Fig. 4f, region ii). Nonsparse test genes that are not well predicted had predicted patterns that were sparser than Visium measurements, suggesting that the disagreement might have been due to dropouts in the snRNA-seq data (Fig. 4g). Finally, about 80% of the transcriptome measured in Visium was highly sparse (Fig. 4f, region iii); the same genes were also too low to be detected by the Allen ISH atlas. This raises the possibility that our predictions may provide more accurate estimates of the real spatial expression for such genes. Supporting this, we compared our predictions with measurements for the two genes available in both MERFISH and our sparse genes. In both cases, our predicted spatial patterns agreed with MERFISH measurements (Fig. 4h).

**Extended Data Fig. 2:**
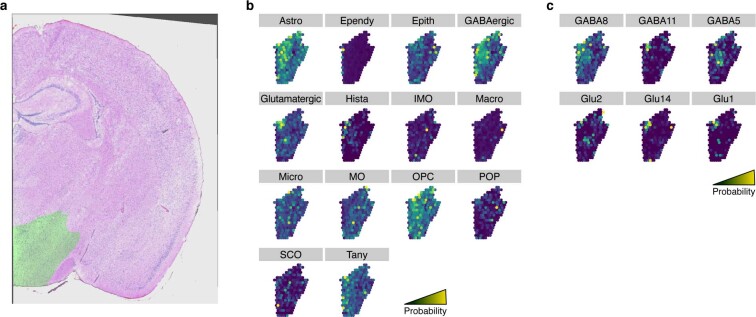
Tangram reveals structural organization in the mouse hypothalamus. **a**. Registration pipeline identifies hypothalamus ROI on Visium section. Nissl-stained image of *Section 1* from the Visium dataset (as in Fig. 4), marked with the hypothalamus ROI (light green) identified by our registration pipeline. **b**. Probabilistic mapping of whole mouse hypothalamus snRNA-seq^32^ to Visium hypothalamus ROI. Probability of mapping (color bar) of each cell type (grey labels; annotations as in^32^) to the Visium hypothalamus ROI. **c**. Probabilistic maps recover neuronal sub-structures in the hypothalamus. Probability of mapping (color bar) of each inhibitory (GABA labels) and excitatory (Glu labels) neuron subsets. Annotations follow^32^.

Notably, Tangram was readily applicable to other brain regions, as we have shown by mapping scRNA-seq data from the mouse hypothalamus^32^ with the hypothalamus in section 1 of our Visium dataset, identified using our registration pipeline (see below; Extended Data Fig. 2a). The resulting predicted cell-type patterns are consistent with expectations (Extended Data Fig. 2b): for instance, ependymal cells and tanycytes are mapped next the third ventricle, and GABAergic and glutamatergic neurons form expected^32^ intricate substructures (Extended Data Fig. 2c). Notably, this mapping was between data that were imperfectly matched, with scRNA-seq collected from the whole hypothalamus and Visium profiling a single coronal slice restricted to a 10-μm-thick posterior, thus containing only a subset of cell types of the entire hypothalamus.

### Spatial localization of chromatin-accessibility patterns with SHARE-seq

**Fig. 5:**
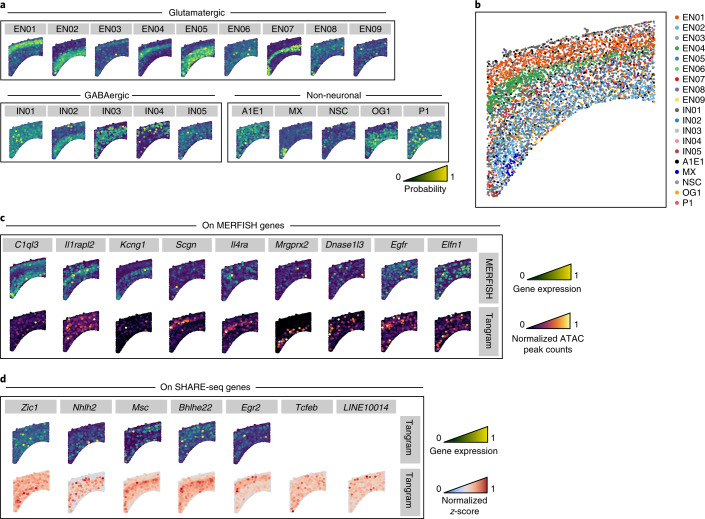
Tangram mapping of multi-omic SHARE-seq profiles yields spatial patterns of chromatin accessibility and transcription factor activity. **a**, Probabilistic mapping of SHARE-seq profiles on MERFISH data. Probability of mapping (color bar) of each cell subset (gray labels) in each of three major categories based on the RNA component of SHARE-seq profiles. **b**, Deterministic mapping. MERFISH slide with segmented cells (dot) colored by the cell-type annotation of the most likely SHARE-seq (RNA) profile mapped on that position by Tangram (legend). **c**, Predicted chromatin-accessibility patterns. MERFISH-measured expression (top; color bar, normalized fluorescence, see Methods) and Tangram-predicted chromatin accessibility (bottom; color bar, normalized reads-in-peak count, see Methods) of select genes (gray labels). **d**, Predicted transcription factor activity patterns. Tangram-predicted expression (top; color bar, mRNA counts) and activity-normalized *z-*score patterns (as inferred from snATAC-Seq, see Methods) (bottom; color bar, dimensionless) of select genes (gray labels) measured only by SHARE-seq.

We next used Tangram’s successful spatial mapping through RNA as a scaffold to map additional molecular profiles with no available spatial data, but that were measurable by single-cell multi-omics. In particular, we set to map joint single-cell RNA expression and assay for transposase-accessible chromatin with sequencing (ATAC-seq) data, which we profiled simultaneously in >3,000 cells from whole mouse brain by SHARE-seq^28^ (detecting about 18,000 genes) and annotated as 9 glutamatergic-cell subsets (EN, excitatory neurons), 5 GABAergic cell subsets (IN, inhibitory neurons), and 5 non-neuronal subsets (A1.E1, MX, NSC, OG1, P1)^28^ (no immune cells were captured, and cortical layer subsets were not annotated). We aligned SHARE-seq data to MERFISH data using the snRNA-seq component of each profile, then transferred the single-nucleus ATAC-seq (snATAC-seq) profile of the same cells to space, to visualize inferred spatial patterns of chromatin accessibility and transcription factor motif scores at single-cell resolution (Fig. 5).

We mapped SHARE-seq data both probabilistically (Fig. 5a) and deterministically (Fig. 5b) and obtained cell-type distributions. Our mapping reveals that EN01s are localized in layer L2/3, EN04s in layer 4, EN07s in layer 5/6, EN05s in layers 5 and 6, and EN02s in layer 6. Interestingly, IN02s seems more prominent in layer 6. Also, the non-neuronal cell type MX (labeled ‘Unconfirmed’^28^) appears to be concentrated at the bottom left part of the ROI, which does not resemble known patterns of cortical cell types. While the mapping is overall consistent, it is less biologically precise than in the previous cases, likely owing to the lack of immune cells (missing ‘puzzle pieces’ for Tangram) and the fact that the cells were not profiled specifically from the cortex.

We used the snRNA-based mapping to infer spatial patterns of chromatin accessibility and transcription factor activity (Fig. 5c,d), and compared them with spatial expression patterns. In some cases, gene expression is higher at a particular cortical layer, but localization is not observed in the projected snATAC-seq (as was the case for *C1ql3*, *Il1rapl2*, and *Kcng1*). In other cases, the projected snATAC-seq forms spatial patterns, even though the corresponding predicted gene does not show a spatial pattern (*Scgn*, *Il4ra*, and *Mrgprx2*). In only a minority of cases, we observed correlation between snRNA-seq and snATAC-seq patterning (*Dnase1l3*, *Egfr*, and *Elfn1*). We similarly visualized inferred spatial patterns of transcription factor motif activity scores (identified from the snATAC-seq profile in each cell^33^) (Fig. 5d). Notably, some exhibited a slightly localized pattern (*Msc*, *Bhlhe22*, and *Egr2*), even for transcription factors whose predicted RNA was neither measured in MERFISH nor in SHARE-seq (for example, *Tcfeb* and *Foxl1* (*LINE10014*)).

### Tangram helps detect cell-type patterns conserved across species

**Extended Data Fig. 3:**
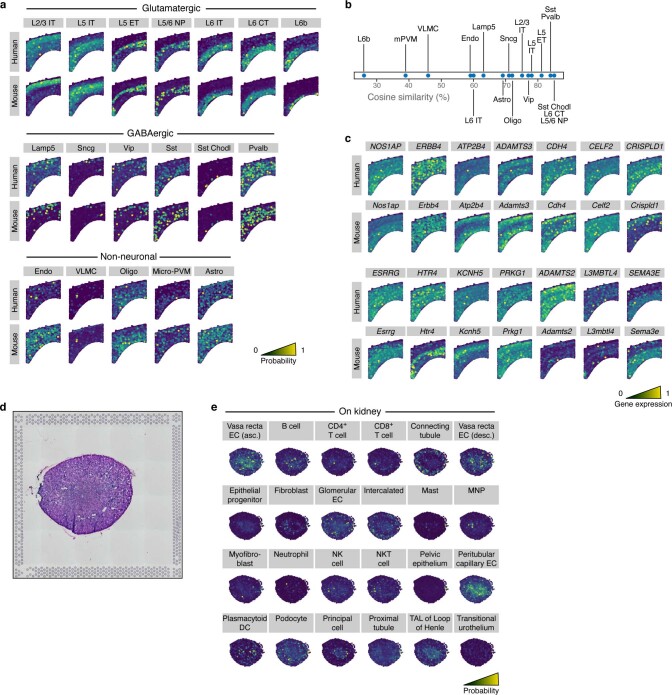
Cross-species mapping of human sc/snRNA-seq to mouse spatial data in brain and kidney. **a-c**. Cross species probabilistic mapping between human primary motor cortex (MOp) snRNA-Seq, and mouse MOp MERFISH. **a**. Agreement in cell type patterning in human-mouse and mouse-mouse mapping. Probability of mapping (color bar) of each cell type (columns) in the cross species case (row label ‘Human’) versus the within-species case (row label ‘Mouse’; probability maps as in Fig. 2a). **b**. Quantitative comparison of cell type patterns between cross and within species mappings. Cosine similarity (blue dots) of cross-species and within-species probability maps for each cell type (labels). **c**. Similarities and differences of individual gene maps between cross and within species mappings. Gene expression (color bar, normalized mRNA counts, Methods) for various genes (horizontal labels) for the cross-species mapping (row label ‘Human’) and the within-species mapping (row label ‘Mouse’). **d,e**. Cross species probabilistic mapping between human kidney scRNA-Seq and mouse kidney Visium. **d**. Hematoxillin&Eosin (H&E)-stained image of a coronal section of mouse kidney on a Visium slide. **e**. Probability of mapping (color bar) of each human kidney cell type on the mouse Visium section.

We next tested how Tangram performs when the input scRNA-seq and spatial data are derived from different species (Extended Data Fig. 3), which we tested in the brain (human MOp snRNA-seq (https://portal.brain-map.org/atlases-and-data/rnaseq/human-m1-10x) and mouse MOp MERFISH) and kidney (human scRNA-seq^34^ and mouse Visium (https://support.10xgenomics.com/spatial-gene-expression/datasets/1.1.0/V1_Mouse_Kidney)) (Supplementary Material). For brain, we found high concordance with same-species mapping for all but two cell types that were absent from human snRNA-seq (Extended Data Fig. 3a,b and Methods), and good but lower similarity at the level of individual genes (Extended Data Fig. 3c). For kidney, the projected cell-type maps (Extended Data Fig. 3d,e) correctly capture several structures and colocalization patterns, whereas some immune-cell types did not map as well, possibly reflecting lower conservation of markers in immune cells.

### A learned histological, anatomical, and molecular atlas of the somatomotor mouse cortex at single-nucleus resolution

**Extended Data Fig. 4:**
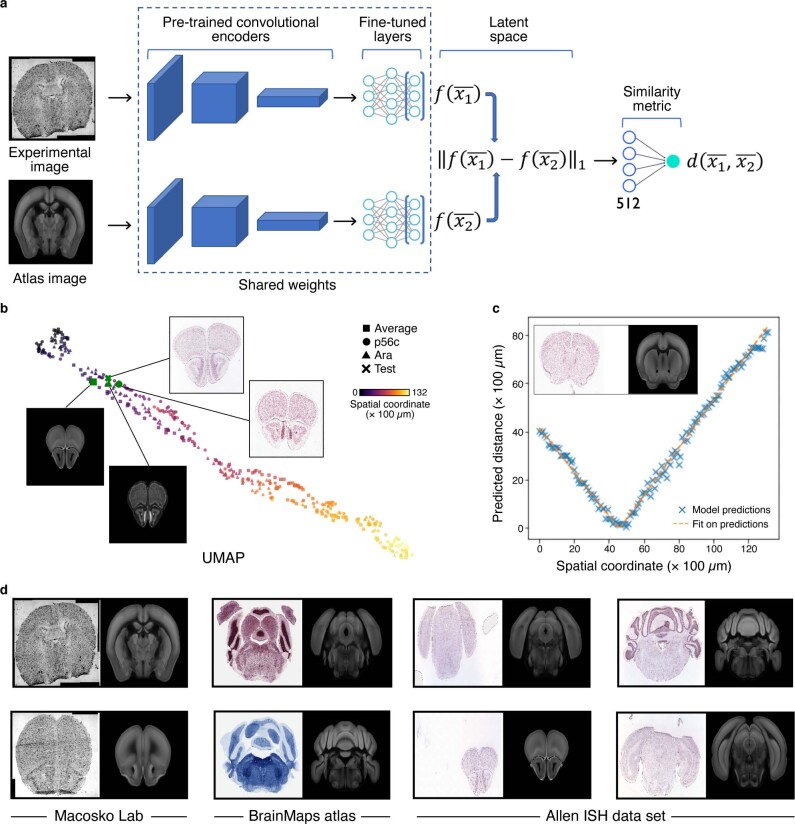
A Siamese network model learns a similarity metric for brain sections based on anatomical landmarks in mouse brain images. **a**. Schematic of neural network architecture. A pair of images is fed to two convolutional encoders, which encode them into a 512-dimensional latent space. The image pair is labeled by the spatial coordinate (*i.e*., coronal depth) difference between the two images. **b**. The learned latent space is a 1D-manifold ordered by spatial coordinates. UMAP plot of the encoded training images from individual atlases (legend) colored by spatial depth (color bar). Insets illustrate four anatomically similar images from three different atlases and a test image. **c**. Prediction of spatial coordinates for a test image. **c**. Predicted spatial coordinate distance (*y* axis) between a test image (inset, left panel) and each image of the training set obtained at different spatial coordinates (*x* axis). Dashed orange line: $\left| {ax + b} \right|$ fit via mean square error minimization $(a\sim - 0.96,\,b\sim 43)$. The minimum of the fit is the predicted spatial coordinate (associated image is in the inset, right panel). **d**. Examples of model predictions (right) on test images (left) from the Macosko lab (first column; Methods), BrainMaps atlas (second column) and Allen ISH dataset (third and fourth columns).

**Extended Data Fig. 5:**
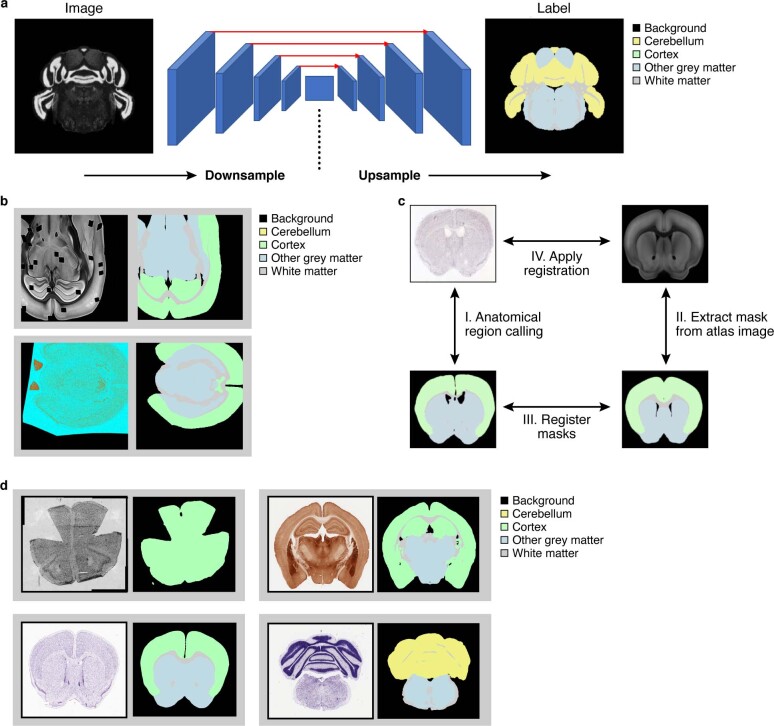
Anatomical region calling via semantic segmentation. **a**. Neural network model used for semantic segmentation. A U-net model is trained on mouse brain images from Allen atlas (left) to recognize five different classes on a mouse brain image (color legend, right). **b**. Augmentation pipeline. Each image undergoes a series of stochastic transformations including affine displacements, dropouts and color shifts (Methods). Four training samples are shown. **c**. Schematic of registration strategy. A segmentation mask of an experimental image is produced (I), the mask of each atlas image is extracted in parallel (II), the two masks are registered to each other (III); and finally the learned transformation is used to register the original images (IV). **d**. Prediction examples. Test images (left) and their predicted anatomical region calls (right).

To demonstrate the integration of molecular and anatomical features, we developed an additional module in Tangram to connect across scales by registering histology/spatial data on an anatomically annotated common coordinate framework (CCF)^35^, such as the Allen CCF for the adult mouse brain^36^. As an alternative to methods that either require supervision or intact tissue, we combine a Siamese neural network model (Extended Data Fig. 4) with a semantic segmentation algorithm (Extended Data Fig. 5) to produce full segmentation masks of anatomical images. The Siamese network model learns a latent space that uniformly encodes irrespective of technical artifacts in the images (such as ‘holes’ in regions predissected for snRNA-seq). The semantic segmentation model produces a segmentation mask that is compatible with the Allen ontology. Because we produce a matching mask, we can automatically register our and the atlas images without providing corresponding landmarks (Methods).

**Fig. 6:**
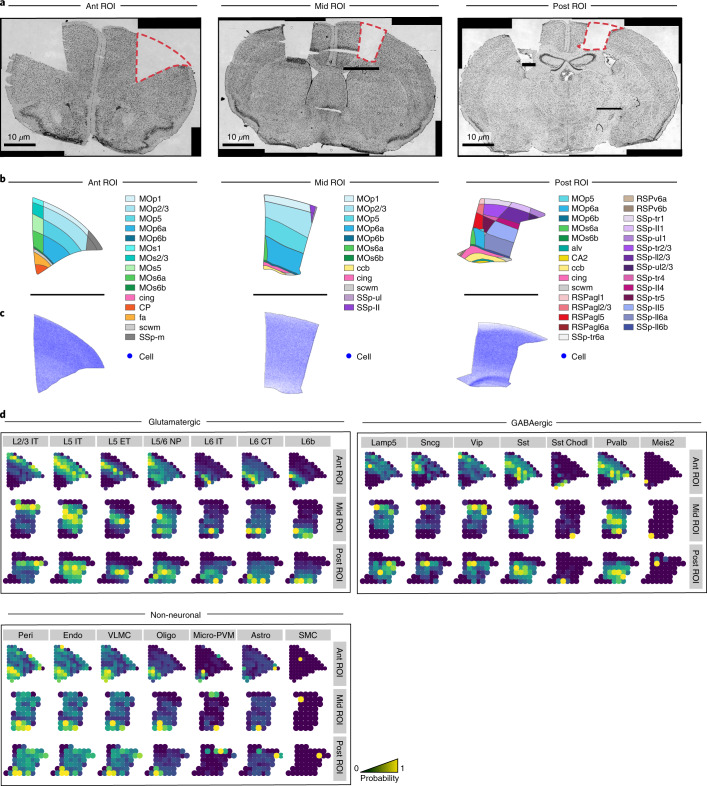
Tangram mapping of snRNA-seq profiles to histological and anatomical mouse brain atlases. **a**, ROIs. Nissl-stained images of coronal mouse brain slices highlighting the three regions of interest (anterior (left), mid (center), posterior (right)) from which snRNA-seq data from the motor area were collected. **b**,**c**, The registration pipeline generates anatomical region and cell-density maps. Anatomical region (**b**, color legend, from the Allen Common Coordinate Framework) and cell map (**c**, color bar, from the Blue Brain Cell Atlas) maps of each of the three dissected ROIs. **d**, Probabilistic mapping of snRNA-seq data on the ROI. Probability of mapping (color bar) of each cell subset (gray label) from each of three major categories within each ROI (rows).

**Extended Data Fig. 6:**
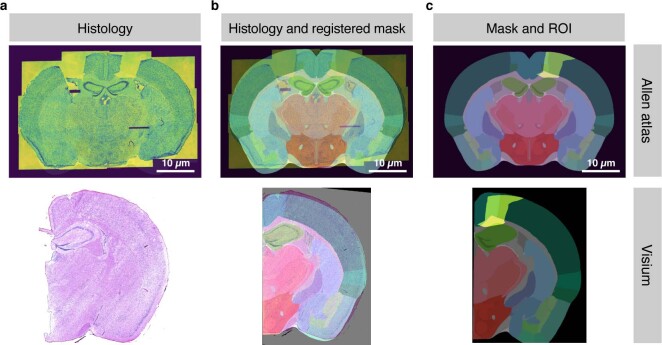
*Post ROIs* registration of Visium histology to the Allen Brain Atlas. **a**. Histological image input. Nissl-stained mouse brain images used to map the *Post ROI* on the Allen Atlas (top; as in Fig. 6a) and on Visium (bottom; as in Fig. 4a). **b**. Mask registration. Histological images from (a) overlaid with the anatomical masks of matching region in the Allen CCFv3. Color legend for anatomical regions as in the Allen Brain Atlas). **c**. *Post ROI*. Sections (as in a) with anatomical labels (as in b) with the post ROI (light green area) identified on the Allen CCFv3 anatomical masks, as a result of registration.

We applied Tangram’s anatomical mapping module to the histological images containing the punch section from which we collected the approximately 160,000 snRNA-seq profiles (Fig. 6a). Using the registration pipeline above, we precisely located the region of dissection on the Allen CCF (Fig. 6b), then queried the Allen Mouse Atlas to estimate spatial gene expression at 200-μm resolution and the Blue Brain Cell Atlas to compute the expected cell density in each spatial voxel (Fig. 6c). We repeated this procedure for the three ROIs, and finally mapped the snRNA-seq profiles to their corresponding ROIs. Note that we used the same pipeline to select the ROI for mapping snRNA-seq profiles onto the histological section measured by Spatial Transcriptomics (Fig. 4a), which was collected at a posterior close to that of the histological section containing the Post ROI (Extended Data Fig. 6). The mapping predictions for cell types across the three ROIs were self-consistent, albeit less accurate than mappings using the higher resolution spatial technologies (Fig. 6d). Cortical layers were successfully recovered across the three ROIs, but L5 ITs and L5/6 NPs display a lower level of localization than in the other cases. GABAergic neurons predictions are biologically sound, and we observed a more concentrated presence of Vip^+^ and Lamp5^+^ cells in the upper layers, as observed with Visium-based mapping. Non-neuronal predictions did not recover sparse mPVM and overly concentrated Peri, Endo, and VLMC cells in the subcortical part. Overall, our mapping results confirmed that glutamatergic-cell-type patterning is simpler to reconstruct than are sparse, granular, cell-type patterns typical of non-neuronal cell types, the latter requiring finer signals from modern spatial technologies.

## Discussion

Genes in organs are expressed in spatially organized patterns at different scales, and understanding these patterns is central to unraveling biological function. Spatially resolved transcriptomic data provide an opportunity to reveal such patterns, but are currently limited by spatial resolution or the number of genes that are measured, and connecting them to other levels or organization can require substantial experimental efforts and expert review. Here, we developed a computational framework, Tangram, to harmonize sc/snRNA-seq data with in situ, histological, and anatomical data, toward a high-resolution, integrated atlas.

Tangram tackles various scenarios by aligning snRNA-seq data onto different spatial data, from high-resolution MERFISH and STARmap, through mid-resolution Spatial Transcriptomics, and to ISH associated with histological and anatomical coordinates. In each case, we validated the computational alignments by recovering consistent spatial maps of cell types and predicting the expression of holdout genes. We applied Tangram primarily in the cortex, but it also performs well in the hypothalamus, which has different kinds of spatial patterns.

Each spatial-measurement modality benefits from different aspects of Tangram: for high-resolution targeted data (smFISH, MERFISH, and STARmap), Tangram expands from signature to genome-wide patterns; for lower resolution spatial data (Visium), Tangram yields single-cell resolution; for datasets with high gene throughput but lower accuracy (STARmap and Visium), Tangram detects and corrects low-accuracy expression patterns; and for multimodal single-cell profiles (SHARE-seq), Tangram uses one modality to generate spatial patterns for the other, yielding spatial multimodal maps. Finally, histology allows registration to the Allen CCF and integration between the cellular and the anatomical scale.

With the notable exception of probabilistic cell mapping (Fig. 4b), Tangram required knowledge on (segmented) cell numbers to perform deconvolution and for mapping on targeted in situ data. Tangram assumed that cells are segmented in preprocessing, which we performed here with dedicated external tools (for example, ilastik (http://www.ilastik.org) or nucleAIzer^37^). However, in higher-density tissues, such as embryos^38^ or tumors, cell-segmentation methods may not perform as well. Future extensions could jointly learn cell segmentation and mapping, as was done in a recently proposed Bayesian method^39^.

In our analyses, a few hundred marker genes, stratified across cell types, sufficed to map the mouse brain cortex transcriptome-wide, observing consistent cell-type patterns in all cases. Notably, although cell mapping can rely on even fewer genes (that is, 22 genes in smFISH; Fig. 1b,d), we could not successfully predict transcriptome-wide spatial gene expression in that case, in contrast to our success with MERFISH (254 genes measured). This suggests that at least a few hundred marker genes could be required to comprehensively map the mouse brain cortex, at least for cell types. As we expand to other more transient cell states and programs, the optimal number of marker genes required for mapping might also depend on the structure of other gene programs and their inter-relations. Tangram can help assess the extent of markers needed to capture a response.

Future applications could use Tangram to discern between biological conditions, leveraging the fact that the Tangram loss function will converge on a smaller value for matching scRNA-seq and spatial datasets. This strategy is compelling in cross-species mappings, in which we recovered conserved patterns for most cell types and genes (Supplementary Material and Extended Data Fig. 3), or in cross modality mapping, such as aligning scRNA-seq data onto spatial proteomic data, to assess the impact of translational and post-translational effects.

When multimodal single-cell profiling data are available, but only one modality is available in the spatial scaffold, Tangram can use it to resolve spatial patterns of the other modality, as we demonstrated using SHARE-seq data to predict spatial patterns for scATAC-seq data. This approach can be adopted with other multimodal single-cell methods (for example, cellular indexing of transcriptomes and epitopes by sequencing^40^ and Patch-seq^41^) or with independently measured single-cell modalities integrated in a common latent space^24,42,43^. This is particularity intriguing in cases for which there is no assay for spatially resolving data of a certain modality. For example, chromatin accessibility could not be spatially resolved at the single-cell level until very recently^44^.

Finally, although our work focused on the mouse brain, Tangram is applicable to other organs, as well as disease tissue. For full integration across scales, Tangram’s registration pipeline requires a CCF, which is currently available for a few organs, and is most advanced for the mouse brain^36^. However, efforts are underway to construct analogous reference maps for different organs^35^, toward the construction of cell atlases of all organs in mice and humans.

## Methods

### Tangram mapping algorithm

#### Introduction

We use the index *i* for cells (that is, snRNA-seq data), *k* for genes, and *j* for spatial voxels (circular spots, pucks, etc.). Our goal is to learn a spatial alignment for the cells, organized as a matrix *S* with dimensions $n_{cells} \times n_{genes}$, where *n*_cells_ is the number of single cells and *n*_genes_ is the number of genes, such that $S_{ik} \ge 0$ is the expression level of gene *k* in cell *i*. In order to map, we voxelize the spatial volume at the finest possible resolution (which depends on the mapping case, for example 200 µm when mapping with the Allen Brain Atlas, individual cells when mapping with MERFISH, and so on), and index the voxels in an arbitrary one-dimensional fashion. We then introduce two quantities: the $n_{voxels} \times n_{genes}$ gene-expression matrix *G*, where $G_{jk} \ge 0$ denotes the expression of gene *k* in voxel *j* (we do not assume that *G* and *S* measure gene expression using the same unit of measures), and a $n_{voxel}$-long vector $\vec{\mathbf{{d}}}$ of cell densities, where $0 \le d_j \le 1$ is the cell density in voxel *j*, and $\mathop {\sum }\limits_j^{n_{voxel}} d_j = 1.$

We aim to learn a mapping matrix *M* with dimension $n_{cells} \times n_{voxels}$, such that $M_{ij} \ge 0$ is the probability of cell *i* of being in voxel *j*. Therefore, we require a probability constraint $\mathop {\sum }\limits_j^{n_{voxel}} M_{ij} = 1$. Our mapping strategy is probabilistic, perform a soft assignment. From the mapping matrix *M*, we further define two quantities: *M*^*T*^*S*, the spatial gene expression as predicted by the mapping matrix, and the vector ${\vec{\mathbf{m}}}$ with components $m_j = \mathop {\sum }\limits_i^{n_{cells}} M_{ij}/n_{cells}$ for the predicted cell density in voxel *j*. Finally, we define the softmax function along the voxel axis for any given matrix $\tilde M$ (with dimensions $n_{cells} \times n_{voxels}$). The resulting matrix *M* has elements:

$$M_{ij} = softmax(\tilde M)_{ij} = \frac{{e^{\tilde M_{ij}}}}{{\mathop {\sum }\nolimits_l^{n_{voxels}} e^{\widetilde {M_{il}}}}}.$$

By applying the softmax, we ensure that $0 \le M_{ij} \le 1$ and $\mathop {\sum }\limits_j^{n_{voxel}} M_{ij} = 1$.

#### Mapping algorithm

To learn the mapping matrix, we minimize the following objective function with respect to $\tilde M$ (note that in the objective we use $M = softmax(\tilde M)$):

1 $$\begin{array}{l}{\Phi}\left( {\tilde M} \right) = KL\left( {\vec{\mathbf{{m}}},{\vec{\mathbf{{d}}}}} \right) - \mathop {\sum }\limits_k^{n_{genes}} cos_{sim}\left( {(M^TS)_{ \ast,k},G_{ \ast,k}} \right) \\\qquad\quad\quad\quad- \mathop {\sum }\limits_j^{n_{voxels}} cos_{sim}\left( {(M^TS)_{j, \ast },G_{j, \ast }} \right),\end{array}$$

where *KL* indicates the Kullback–Leibler divergence, cos_sim_ is the cosine similarity function, and * indicates matrix slicing. The first term is the density term: we enforce that the learned density distribution is as similar as possible to the expected density. The second term is the gene/voxel expression term: it enforces that the predicted expression for each gene over the voxels is proportional to the expected gene expression over the voxels. The third term is the voxel/gene expression term: for each voxel, the predicted gene expression needs to be proportional to the expected gene expression. Optionally, we can also activate an entropy regularizer to minimize the entropy of the spatial distribution of each cell, to ensure that the spatial probabilities of each cell are peaked over a narrow portion of space. (In practice, we did not need to use this feature, as all probabilities were peaked in all cases analyzed in this study).

Minimization is obtained via gradient-based optimization using the PyTorch library.

Using the objective (1), Tangram maps all sc/snRNA-seq profiles onto space. If the number of sc/snRNA-seq profiles is higher than the known number of cells in the spatial data, Tangram can instead filter the sc/snRNA-seq profiles and learn the optimal subset of sc/snRNA-seq profiles that best explains the spatial data. The latter approach is explained next.

#### Mapping with a filter

We introduce a filter vector ${\vec{\bf f}}$ of dimension *n*_cells_ so that cell *i* can either be mapped (*f*_*i*_ = 1) or not mapped (*f*_*i*_ = 0). To filter, we multiply each row of the single-cell matrix, $S_{i, \ast },$ and each row of mapping matrix, $M_{i, \ast },$ by *f*_*i*_, as shown below in the new objective. The filter values *f*_*i*_ are learned during training, in order to retain the cells that best explain the spatial data. To explicitly promote Boolean values (that is, 0s or 1s) in the filter values, we add a filter regularizer in the objective. To enforce the total number of filtered cells, we introduce a count term: a soft constraint in the objective that promotes a number of mapped cells in the filter equal to $n_{target\_cells}$. With this in mind, we formally define the objective. We define a real vector ${\vec{\tilde{\bf f}}}$ of dimension *n*_cells_ and define ${f_i} = \sigma ({\tilde f}_i)$, where we apply the sigmoid function *σ* to ensure that $0 \le f_i \le 1$. We then define $S^f = diag({\vec{\bf f}}) \cdot S$ and $M^f = diag({\vec{\bf f}}) \cdot M$, namely, the filtered versions of the single cell matrix and the mapping matrix, respectively. We also define ${\vec{{\bf{m}}^{\bf{f}}}}$, a vector with components $m_j^f = \mathop {\sum }\limits_i^{n_{cells}} M^f/\mathop {\sum }\limits_i^{n_{cells}} f_i$, as the predicted density of filtered cells in voxel *j*. The objective function, which we minimize with respect to $\tilde M$ and $\overrightarrow {\tilde f}$, is:

2 $$\begin{array}{l}{\Phi}\left( {\tilde M,\,\overrightarrow {\tilde f} } \right) = KL\left({\vec{{\bf{m}}^{\bf{f}}}}, {\vec{\bf d}} \right) - \mathop {\sum }\limits_k^{n_{genes}} cos_{sim}\left( {(M^TS^f)_{ \ast,k},G_{ \ast,k}} \right)\\ - \mathop {\sum }\limits_j^{n_{voxels}} cos_{sim}((M^TS^f)_{j, \ast },G_{j, \ast }) - \lambda _{r_1}\mathop {\sum }\limits_{i,j}^{n_{cells},\,n_{voxels}} M_{ij}log\left( {M_{ij}} \right)\\ + abs(\mathop {\sum }\limits_i^{n_{cells}} f_i - {{{\mathrm{n}}}}_{{{{\mathrm{target}}}}\_{{{\mathrm{cells}}}}}) + \mathop {\sum }\limits_i^{n_{cells}} (f_i - f_i^2).\end{array}$$

The fourth term corresponds to the entropy regularizer, and the last two terms correspond to the count term and the filter regularizer, respectively.

#### Annotations transfer

The output of the mapping algorithm is the learned mapping matrix *M* (with, optionally, the learned filter ${\vec{\mathbf{f}}}$). Once the mapping outcome is computed, any kind of annotation can be transferred from the sc/snRNA-seq data onto space.

We define *A* as the annotation matrix with dimensions $n_{cells} \times n_{annotations}$, where $n_{annotations}$ is the number of different annotations characterizing single cells (for example, genes, cell types, or any other modality). If annotations are categorical values (such as cell types), we one-hot encode them over multiple columns in *A*. Annotations in space are retrieved by computing:

$$A_{transf} = M^TA,$$

or, if the filter has also been learned, via:

$$A_{transf}^f = M^T(diag\left( {\vec{\mathbf{f}}} \right) \cdot A).$$

The computed matrix $A_{transf}$ has dimensions $n_{\rm{voxel}} \times n_{\rm{annotations}}$, and therefore denotes the annotations in space.

#### Cell-type calling

When *A* describes cell types, $A_{transf}$ describes the probabilistic counts for each cell type in each cell voxel. This corresponds to probabilistic mapping and can be interpreted as the mixture of cell types that best explain the in situ gene expression. When the technology allows for single-cell spatial resolution, voxels correspond to single cells in space. In this case, probabilistic mapping can be seen as an unnormalized probability distribution over cell types for the voxel or cell. As a consequence, for technologies with single-cell spatial resolution, we can define a deterministic mapping as the mapping assigning the most likely cell type in this distribution to each spatial cell.

#### Mapping spatial data from targeted technologies

smFISH (Fig. 1), MERFISH (Fig. 2), and STARMap (Fig. 3) allow for single-cell spatial resolution; therefore, the number of spatial voxels needs to be equal to the number of cells. As snRNA-seq profiles are typically more numerous than are MERFISH voxels, we can use the mapping with the learned filter, namely, Eq. (2). In this case, $n_{target\_cells} = n_{voxel}$ and the expected density ${\vec{\mathbf{d}}}$ is uniform and equal to $d_j = \frac{1}{{n_{voxel}}}$ for all *j*. This enforces that each cell is mapped to one voxel only and vice versa. If the number of available single cells is lower than the number of spatial spots, we can instead map with Eq. (1), using the same constant density ${\vec{\mathbf{d}}}$.

For the MERFISH case, we mapped 58,022 10Xv3 snRNA-seq profiles in 4,951 spatial spots. From the 26,944 genes in the snRNA-seq data, we selected 1,408 marker genes as the top 100 marker genes stratified across the 22 cell types. We mapped using the intersection between these marker genes and the 254 MERFISH genes, which corresponded to 253 genes. For the leave-one-out validation strategy, we partitioned the genes into 252 training genes and a single test gene (unseen in the learning of the model), and repeated the training 253 times, each time leaving out a different gene, to obtain a prediction for each gene.

For the smFISH case, we mapped 11,759 SMART-Seq2 snRNA-seq profiles in 4,840 spatial spots. In this case, 40,056 transcripts are measured in the snRNA-seq data. Only 22 genes were measured in smFISH, all of which were also present in the snRNA-seq data. Therefore, we used all 22 genes for mapping.

For the STARmap case, we used the same snRNA-seq data as for smFISH, which we mapped on 1,550 spatial spots. We took the intersection of 995 genes between the 1,020 STARmap transcripts and the 40,056 transcripts in the snRNA-seq data. We used these 995 genes for mapping.

The algorithm converges after 1,200 epochs in all the experiments. Tangram’s mapping output is always probabilistic. For deterministic mapping, we start from a probabilistic mapping and then choose the highest probability cell in each spatial voxel.

#### Mapping Visium data

We began by identifying the Post ROI on the Visium histological image using our registration pipeline (below). Next, we segmented the cells within the ROI using the software ilastik (https://www.ilastik.org). We then built the density vector ${\vec{\mathbf{d}}}$, by computing the cell density inside each voxel (that is, Visium circle, as in Fig. 1e). We mapped using the objective described in Eq. (1). Mapping yields the matrix *M*, which we used to generate probability maps for the cell types within the ROI. To deconvolve, we mapped using Eq. (2), to constrain the expected number of cells in each Visium voxel. Specifically, we used $n_{target\_cells} = n_{seg}$, where $n_{seg}$ is the total number of segmented cells in the Visium ROI, to enforce that only a subset of cells is actually mapped. The count term combined with the density term led to the expected number of mapped cells in each Visium voxel. After training, we assigned the types of the cells mapped within each voxel randomly to specific segmented cells within that voxel.

For probabilistic mapping on Visium data, we ran the optimizer for 300 epochs to reach convergence. At the end, more than 99% of cells were assigned to an individual voxel with probability greater than 50%. For deterministic mapping in deconvolution, we trained the optimizer for 6,000 epochs to reach convergence. At the end, more than 99% of cells were assigned to an individual voxel with probability greater than 50%. For the section 1 dataset, the number of cells filtered ($f_i > 0.5$) was 880 (89% of segmented cells). Segmented cells for which there was no filtered mapped cell are not shown in the figures.

For both probabilistic and deterministic mapping, we used 58,022 10Xv3 snRNA-seq profiles for 162, 161, and 134 spatial spots, respectively, in section 1, section 2, and section 3. Among the 26,944 transcripts in the snRNA-seq data, 1,408 marker genes were selected. We mapped using the intersection of these genes with Visium genes (31,053), corresponding to 1,408 genes.

Finally, cell segmentation is required for the method for deconvolving Visium data. However, Tangram does not require cell segmentations for obtaining probability maps of cell types (Fig. 4b) or correcting gene expression (Fig. 4e). Tangram does not currently perform cell segmentation, for which we used pre-existing tools, such as ilastik (http://www.ilastik.org). and nucleAIzer^37^. Both tools were used to segment the histological images of our Visium dataset, and final segmentation was obtained by merging the results from the two methods.

#### Mapping Allen atlas data

We used 58,022 10Xv3 snRNA-seq data for 83, 38, and 43 spatial spots, respectively, in the anterior, mi,d and posterior ROIs. Among 26,944 transcripts in the snRNA-seq data, 1,408 marker genes were selected. We mapped using the intersection between these genes with Allen atlas genes measured coronally (overall, 4,345 genes); the intersection corresponds to 750 genes. The algorithm converged after 150 epochs.

### Data collection—snRNA-seq data and histological images

#### Mouse experiments

Mice were group housed with a 12-hour light–dark schedule and allowed to acclimate to their housing environment for 2 weeks post-arrival. All procedures involving animals at MIT were conducted in accordance with the US National Institutes of Health Guide for the Care and Use of Laboratory Animals under protocol number 1115-111-18 and were approved by the Massachusetts Institute of Technology Committee on Animal Care. All procedures involving animals at the Broad Institute were conducted in accordance with the US National Institutes of Health Guide for the Care and Use of Laboratory Animals under protocol number 0120-09-16.

#### Brain preparation prior to anatomical dissection and snRNA-seq

At 60 days of age, C57BL/6J mice (50% males, 50% females) were anesthetized by administration of isoflurane in a gas chamber, with a flow of 3% isoflurane for 1 minute. Anesthesia was confirmed by checking for a negative tail pinch response. Animals were moved to a dissection tray and anesthesia was prolonged via a nose cone through which 3% isoflurane flowed for the duration of the procedure. Transcardial perfusions were performed with ice-cold pH 7.4 HEPES buffer containing 110 mM NaCl, 10 mM HEPES, 25 mM glucose, 75 mM sucrose, 7.5 mM MgCl_2_, and 2.5 mM KCl to remove blood from the brain and other organs sampled. The brain was removed immediately and frozen for 3 minutes in liquid nitrogen vapor and moved to long-term storage at –80 °C. A detailed protocol is available at protocols.io (https://www.protocols.io/view/fresh-frozen-mouse-brain-preparation-for-single-nu-bcbrism6).

#### Generation of MOp dissectates and snRNA-seq data

Frozen mouse brains were securely mounted by the cerebellum onto cryostat chucks with OCT embedding compound such that the entire anterior half, including the primary motor cortex (MOp), was left exposed and thermally unperturbed. Dissection of 3 consecutive 500-μm anterior–posterior (A–P) spans of the MOp was performed by hand in the cryostat using an ophthalmic microscalpel (Feather safety Razor no. P-715) precooled to –20 °C and donning 4× surgical loupes. Each 500-μm step was accomplished by advancing the cryostat (Leica CM3050S) 100 μm 5 times in trimming mode and cutting out each dissectate 100 μm at a time. This stepwise approach serves to ameliorate disruption of the brain tissue surface that occurs with large steps. Each excised tissue dissectate pool was placed into a precooled 0.25-ml PCR tube using precooled forceps and stored at –80°C. In order to assess dissection accuracy, 10-μm coronal registration sections were taken at each of the 500-μm A–P dissection junctions and imaged following Nissl staining. Nuclei were extracted from the frozen tissue dissectates using gentle, detergent-based dissociation, according to a protocol (https://www.protocols.io/view/frozen-tissue-nuclei-extraction-bbseinbe) adapted from one generously provided by the McCarroll lab, and loaded into the 10x Chromium V3 system (10x Genomics). Reverse transcription and library generation were performed according to the manufacturer’s protocol.

#### Analysis of sc/snRNA-seq data

**Extended Data Fig. 7:**
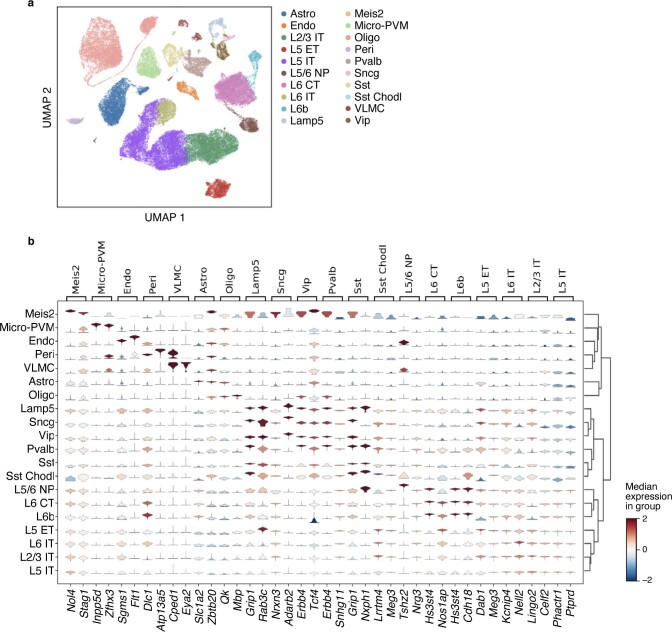
Mouse motor cortex cell subsets based on snRNA-seq. **a**. Cell clusters. UMAP embedding (Methods) of snRNA-seq profiles (dot) colored *post hoc* by cell type clusters (color legends with abbreviations; complete name in Extended Data Table 1). **b**. Cell subset specific markers. Distribution of normalized expression level (z-scores of logarithmic counts, color: median expression; Methods) for the two top marker genes (columns, bottom) of each cell type (rows; columns, top).

All sc/snRNA-seq datasets were analyzed using the scanpy package^45^. All data were preprocessed via the following steps: we removed cells with high mitochondrial gene content and normalized the data to correct for library size. The resulting snRNA-seq data were ready to be mapped with Tangram. To compute marker genes, we applied a computational pipeline described in the tutorial of the scanpy package^46^ (https://scanpy-tutorials.readthedocs.io/en/latest/pbmc3k.html). Briefly, we applied the function $f\left( x \right) = {{{\mathrm{log}}}}(1 + x)$ to the normalized counts, and standardized gene expression. Next, we performed principal components analysis and retained the first 50 principal components of the gene expression matrix and computed a *k*-nearest neighbor (*k*-NN) graph using the Euclidean distance in expression space. All cell types in the sc/snRNA-seq data were preannotated, and we verified via a UMAP plot showing that cells with the pre-existing annotations form distinct clusters in transcriptome space (results shown for MOp snRNA-seq in mouse; Extended Data Fig. 7a). We then identified differentially expressed marker genes by a statistical *t*-test (the top two genes for each cell type for the MOp snRNA-seq in mouse are shown in Extended Data Fig. 7b). In mapping onto spatial transcriptomic data, we chose the top 100 marker genes for each cell type, which overall sums up to ~1,000 genes. We chose not to map using the entire transcriptome, as several genes in Visium data are not high quality (for example, because of dropouts), and it would not be beneficial to add those genes to the training set. Also, by leaving out a large part of the transcriptome, we have a convenient test set of genes. Finally, nonmarker genes fluctuate in their basal signal and would not contribute to mapping.

We used normalized quantities to visualize gene expression via mRNA counts (Figs. 1b,c,f, 2c,e,f, 3c–e, 4e,g,h, and 5c,d), gene expression via fluorescence (Figs. 2c,f, 3c, 4h, and 5c) chromatin accessibility via ATAC peak counts (Fig. 1c,f), and transcription factor activity via *z*-scores (Fig. 5d). Normalization is performed by rescaling the colorbar in each image, so that the minimum (and maximum) value of the image correspond to the color with minimum (and maximum) value in the colorbar. This is the default behavior of the plotting functions of the Python library matplotlib (https://matplotlib.org), which we used throughout the manuscript.

### Data collection—Visium

#### Mice

All mouse work was performed in accordance with the Institutional Animal Care and Use Committees (IACUC) and relevant guidelines at the Broad Institute, with protocol IACUC 0147-02-17.

#### Tissue processing

Fresh-frozen wild-type C57BL/6 whole mouse brain was embedded in OCT (TissueTek Sakura) and cryo sectioned at 10-μm thickness at –20°C. Tissue sections were placed in 6.5-mm squared capture areas on precooled Visium Tissue Optimization slides (3000394, 10x Genomics) and Visium Spatial Gene Expression slides (2000233, 10x Genomics) and were adhered by warming the backside of the slides and placed at –80 °C for up to 3 days.

#### Visium spatial gene expression library generation

The tissue optimization sample slide and spatial gene expression slide were processed according to the manufacturer’s protocols. Briefly, tissue sections were warmed to 37°C for 1 minute and fixed for 30 minutes in ice-cold methanol, followed by 1 minute of incubation in isopropanol at room temperature. Tissues were then H&E-stained according to the protocol. Morphology brightfield images were taken with a Zeiss Axio microscope with the Metafer slide-scanning platform (Metasystems) with a ×10 objective. For the tissue optimization slide fluorescent images, a TRITC filter and ×10 resolution were used. Images were joined together with the VSlide software (Metasystems) and exported as tiff files. To optimize tissue permeabilization time, 6 different time points with 3-minute increments were tested on the tissue optimization sample slide. Twelve minutes of permeabilization was used for the spatial gene-expression slide. RNA released from the tissue was converted to complementary DNA by priming to the spatial barcoded primers on the glass via reverse transcription in the presence of template-switching oligonucleotide, to generate full-length, spatially barcoded, unique molecular identifier (UMI)-containing complementary DNA. Subsequently, following second-strand synthesis, a denaturation step released the cDNA, followed by PCR amplification. Finally, sequencing-ready, indexed spatial gene-expression libraries were constructed. Two of the libraries were pooled together and sequenced on a NextSeq 500/550 High output kit at 1.8 pM concentration. The sequencing settings were: read 1, 28 cycles; read 2, 91 cycles; index 1, 8 cycles.

#### MOp Visium raw read processing

Raw FASTQ files generated by Illumina’s BCL2FASTQ conversion and the histology H&E images were provided as input to the SpaceRanger software (10x Genomics) version 1.1.0, available at https://support.10xgenomics.com/spatial-gene-expression/software/downloads/latest. Sequencing reads were mapped to the mm10 reference mouse genome using STARv2.5 mapping as part of SpaceRanger suite. Spatial barcodes were assigned by SpaceRanger to the barcoded spatial spots and aligned with the tissue image with the aid of the fiducial frames. Barcodes/UMI and genes were counted for the individual spots to generate an output matrix of gene expression per spot that was used as input for downstream data analysis.

#### MOp MERFISH data preprocessing

We preprocessed the MERFISH to remove subcortical cells. To identify subcortical cells, we identify cells overly expressing *Nxph4* (a marker gene of L6b region) and fit those cells with a square-root polynomial. All cells below the fit were considered subcortical and were removed.

### Image datasets for registration pipeline

To locate ROIs, we used images of Nissl-stained coronal mouse brain slices collected in the Macosko lab. To train and test the models presented in Fig. 6 and Extended Data Figs. 4 and 5, we used the following public image datasets:

- (dataset avg)**:** 1,320 images or segmentation masks of coronal slices from the average template of the Allen adult mouse brain atlas at resolution of 10 μm (available at http://download.alleninstitute.org/informatics-archive/current-release/mouse_ccf/average_template/slice_images/).
- (dataset ara): 1,320 images or segmentation masks of coronal slices from the Nissl template of the Allen adult mouse brain atlas at resolution of 10 μm (available at http://download.alleninstitute.org/informatics-archive/current-release/mouse_ccf/ara_nissl/).
- (dataset p56c)**:** 132 images or segmentation masks of coronal slices from the Allen P56 coronal reference atlas (available at https://mouse.brain-map.org/experiment/thumbnails/100048576?image_type=atlas).
- (dataset p56d): 504 images of coronal slices from the Allen Development Atlas P56 (available at http://help.brain-map.org/display/atlasviewer/Allen+Developing+Mouse+Brain+Atlas).
- (dataset brainmaps): 111 images of coronal slices from Nissl-stained BrainMaps atlas (ID: 43) (available at http://brainmaps.org/index.php?action=viewslides&datid=43), and 87 images of coronal slices from Nissl-stained BrainMaps atlas (ID: 38) (available at http://brainmaps.org/index.php?action=viewslides&datid=38).
- (dataset ish): 30 images of coronal slices from the Allen ISH Data (available at https://mouse.brain-map.org/search/index).

#### Siamese network model for depth calling

Building on methods for face recognition, we taught a latent space on mouse brain images using a Siamese network model (Extended Data Fig. 4a). We trained the model (below) so that each image was encoded according to salient anatomical landmarks, whereas technical properties such as illumination or staining were factored out. The learned latent space displayed a one-dimensional manifold structure, where the ‘head’ of the manifold contains images from the olfactory bulb, and the ‘tail’ contains images from cerebellum (Extended Data Fig. 4b). The model predicted the image from the Allen CCF at the same coronal depth of our histological image. We validated the predictions by checking consistency across the entire training set (Extended Data Fig. 4c), and by expert visual inspection (Extended Data Fig. 4d). We then used the trained model to retrieve the image from the Allen CCF onto which we registered our histological image.

We used datasets avg, ara, p56c, and p56d for training. Training images were resized to 224 × 224 and casted to numerical type float32. Pixel values were rescaled between zero and one, prior to training. All images were augmented using the imgaug (https://github.com/aleju/imgaug) library. We used numerical coordinates as training labels, indicating the spatial coronal depth (that is, posterior) of each mouse brain image on a scale of 10 μm. For the avg and ara datasets, labels were readily available from their tensor coordinates. Labels for the p56c and p56d datasets were also readily obtained using the AllenSDK API (https://allensdk.readthedocs.io/en/latest/). Dataset brainmaps and ish were manually annotated and used as test sets.

In designing the Siamese network model, we used a DenseNet169 encoder pretrained on the ImageNet dataset and open-sourced through Keras Applications. We finetuned the encoder by training the last convolutional layer. We added two fully connected layers on top of the encoder in order to map the extracted features to our 512-dimensional latent space. A last fully connected layer was used to map the latent space to the model output as represented in Extended Data Fig. 4. All fully connected layers were trained.

A training sample consisted of two random images from the annotated datasets. The difference in spatial depth coordinates between the two images, denoted by $\widehat {d_i}$, was used as the label. For example, if the first image was at posterior (depth) 500 μm and the second at a posterior 700 μm, the corresponding label would be $\widehat {d_i} = 200$. We used as penalty the mean-squared error between the spatial depth difference predicted by our network d_i_, and the labels $\widehat {d_i}$:

$$MSE(d,\hat d) = \frac{1}{N}\mathop {\sum }\limits_{i = 1}^N (d_i - \widehat {d_i})^2,$$

where *N* indicates the number of training samples. We trained the model for 50 epochs using 18,000 image pairs per epoch, partitioned to batches of 16 images.

#### Semantic segmentation model for anatomical region calling

The goal of the semantic segmentation model is to generate a custom mask for our images using the same color scheme adopted by the Allen ontology. For this, we applied semantic segmentation, and segmented five classes in our histological image (Extended Data Fig. 5a): background, cortex, cerebellum, white matter, and other gray matter. Because the training set is scarce, as described below, we adopted a combination of transfer learning and heavy augmentation during training (Extended Data Fig. 5b) and validated it by inspecting predictions on test atlases (Extended Data Fig. 5d). Finally, we combined segmentation with the Siamese model described above, to obtain a fully automated registration pipeline (Extended Data Fig. 5c), leveraging the fact that registering two masks (one on the Allen image and one on the image of our sample) is a simpler problem than registering the two images directly.

To train the semantic segmentation model, we used datasets avg, ara, and p56c as training sets, since masks were available. Training images were resized to 512×512 and casted to type float32. Pixel values were rescaled between zero and one. As labels, we used superimposable segmentation masks with the same dimension as the training images. Each mask was one-hot encoded into a 5-channel tensor to annotate each pixel into five different classes (Extended Data Fig. 5): background (black), cortex (green), cerebellum (yellow), other gray matter (gray), and white matter (brown). We used colors consistent with the Allen ontology to facilitate registration. For the avg and ara datasets, we used masks from the Allen CCFv3 ontology 2017 (available at http://download.alleninstitute.org/informatics-archive/current-release/mouse_ccf/annotation/ccf_2017/annotation_10.nrrd). For the p56c dataset, we downloaded the SVG masks from the Allen Institute website, and rendered them into images using the library released in this study, which builds on Cairo SVG (https://cairosvg.org). Both images and masks were augmented using the same pipeline adopted for the Siamese model. In transforming the masks, we ensured that the one-hot structure was preserved in the masks after augmentation.

We used a semantic segmentation model from the Tensorflow Keras version of the segmentation_models library (https://github.com/qubvel/segmentation_models). Specifically, we chose a U-NET architecture with a ResNet50 backbone. All weights have been randomly initialized following the He scheme, with the exception of the ResNet50 encoder which was pretrained on ImageNet. The model was trained to optimize the superposition of the cross entropy and Jaccard index (that is, intersection-over-union). The loss function is defined as:

$$L(g,\,p) = - g \cdot \log(p) - \frac{{p\mathop { \cap }\nolimits^ g}}{{p\mathop { \cup }\nolimits^ g}}.$$

Where *g*a is the ground truth image and *p* is the corresponding model prediction. The model last unit employs a softmax activation function, thus outputting the probability of each pixel to be in each of the five classes. By applying an argmax function, we assign each pixel to its most probable class. Finally, we relied on test-time augmentation to increase model performances: each test image was augmented 12 times, and final predictions were deaugmented and averaged.

### Reporting Summary

Further information on research design is available in the Nature Research Reporting Summary linked to this article.

## Online content

Any methods, additional references, Nature Research reporting summaries, source data, extended data, supplementary information, acknowledgements, peer review information; details of author contributions and competing interests; and statements of data and code availability are available at 10.1038/s41592-021-01264-7.

## Supplementary information

Supplementary Information Supplementary Material

Reporting Summary

Supplementary Table

## Data Availability

smFISH data, Visium VISp data, MERFISH VISp data and Smart-Seq2 VISp snRNA-seq data are available at http://github.com/spacetx-spacejam/data. MERFISH MOp data are available at the Brain Image Library (https://doi.brainimagelibrary.org/doi/10.35077/g.21). SHARE-seq dataset are available (GSE140203). The STARmap dataset is publicly available at ref. ^14^. All other data are available at: https://console.cloud.google.com/storage/browser/tommaso-brain-data.

## Code availability

Tangram code is available at https://github.com/broadinstitute/Tangram, along with the datasets used to generate Fig. 1.
