# Supplementary material for: Deep learning and alignment of spatially resolved single-cell transcriptomes with Tangram
Source: Nat Methods. 2021 Oct 28;18(11):1352–62. doi: 10.1038/s41592-021-01264-7 (PMC8566243; doi:10.1038/s41592-021-01264-7)
Supplement: Supplementary file 1 — Supplementary Material [file 41592_2021_1264_MOESM1_ESM.pdf]

nature portfolio

https://doi.org/10.1038/s41592-021-01264-7

Supplementary information

Deep learning and alignment of spatially resolved single-cell transcriptomes with Tangram

In the format provided by the authors and unedited

Deep learning and alignment of spatially-resolved single cell transcriptomes with Tangram – Supplementary Material

Tommaso Biancalani[*,1,9,#], Gabriele Scalia[*,1,10], Lorenzo Buffoni[2], Raghav Avasthi[1,3], Ziqing Lu[1,3], Aman Sanger[1], Neriman Tokcan[1], Charles R. Vanderburg[1], Åsa Segerstolpe[1], Meng Zhang[4,6], Inbal Avraham-Davidi[1], Sanja Vickovic[1], Mor Nitzan[1,5,11] Sai Ma[1,7,8], Ayshwarya Subramanian[1], Michal Lipinski[1,8], Jason Buenrostro[1,8], Nik Bear Brown[3], Duccio Fanelli[2], Xiaowei Zhuang[4,6], Evan Z. Macosko[1] and Aviv Regev[1,6,7,9,#]

[1]Broad Institute of MIT and Harvard, Cambridge, MA 02142,

[2]Department of Physics and Astrophysics, University of Florence, Florence, Italy

[3]Northeastern University, Boston, MA 02115

[4]Department of Chemistry and Chemical Biology, Department of Physics, Harvard University

[5] School of Engineering and Applied Sciences, Harvard University

[6]Howard Hughes Medical Institute

[7]Department of Biology, MIT, Cambridge, MA 02140

[8]Department of Stem Cell and Regenerative Biology, Harvard University, Cambridge, MA, 02138 USA.

[9]Current address: Genentech, 1 DNA Way, South San Francisco, CA, 94080

[10]Current address: Roche, Monza, Italy

[11]Current address: School of Computer Science and Engineering, Racah Institute of Physics, Faculty of Medicine; The Hebrew University, Jerusalem, Israel

* These authors contributed equally

# To whom correspondence should be addressed: tommaso.biancalani@gmail.com (TB) aviv.regev.sc@gmail.com (AR)

Tangram helps detect cell type patterns conserved cross species

Here, we tested how Tangram performs when the input scRNA-seq and spatial data do not match as closely, as in the special case when each is derived from a different species (**Extended Data Fig. 3**), which we tested in both the brain and the kidney (chosen by data availability).

For cross species brain mapping, we mapped pre-annotated human brain MOp snRNA-seq data[1], collected post mortem (**Methods**) onto mouse MERFISH MOp, and compared it to the corresponding within-species mapping (mouse MOp snRNA-seq with mouse MERFISH MOp; **Fig. 2**), finding high concordance in the mapping patterns for most cell types (**Extended Data Fig. 3a,b, Methods**). Specifically, we compared the probability maps for the cell types shared between the human and mouse snRNA-seq datasets, namely, all cell types except *Meis* and *Peri*, which were not present in the human snRNA-seq data (**Extended Data Fig. 3a**). Excitatory neurons (glutamatergic) formed the expected cortical patterns, with the sole exception of L6b neurons being distributed across layer 6, and inhibitory neurons (GABAergic) were also in excellent agreement, with *Lamp5* cells being slightly less localized in the cross-species case. The least similar patterns were in non-neuronal *mPVM* and *VLMC* cell types, also reflected quantitatively by the cosine similarity metric (**Extended Data Fig. 3b**).Overall, the mappings at cell type level were highly concordant, consistent with the high similarity of cell profiles for mouse and human cortical brain cells[2].

When we compared the predicted spatial gene expression of individual genes (focusing on gene orthologs of those we validated against images from the Allen atlas (**Figs. 2e,f**)), the similarity, while present, was lower than that for cell type maps (**Extended Data Fig. 3c**). Interestingly, the

extent of agreement does not relate to specific cell types. For instance, some marker genes of cortical layers display similar patterns (*SEMA3E* and *ADAMTS3*), whereas other cortical markers are not localized in the cross-species case (*HTR4, KCNH5* and *CRISPLD1*). Differences in the extent of agreement are also observed between pairs of genes with granular patterns (*ERBB4 vs ESRGG*) and pairs of paralogs (*ADAMTS2* and *ADAMTS3*).

Tangram also successfully mapped scRNA-seq profiles from human kidney[3] onto a Visium coronal slide for mouse kidney[4] (**Extended Data Fig. 3d, Methods**). To this end, we computed the top 100 marker genes for each pre-annotated cell type in the scRNA-seq data (**Methods**), intersected them with their orthologs measured in Visium, and performed a probabilistic mapping using the shared orthologs as a training set (**Extended Data Fig. 3e**). The projected cell type maps (**Extended Data Fig. 3e**) correctly capture several structures, including the connecting tubule, the collecting duct (the center part of the ROI where *Principal cells* concentrate), and the vasa recta as we move from the cortex to the medulla. We also observe co-localization of podocytes with *Glomerular EC*, and co-localization of *Vasa Recta EC* with *TAL of Loop of Henle* cells in the medulla, as we expected from kidney anatomy. Tangram did not assign high probability to *Pelvic epithelium* cells and *Transitional urothelium* cells, as we expected, since these pelvic regions are not captured in the coronal slice. Some immune cell types (*CD4*$^{+}$ and *CD8*$^{+}$ *T* cells, *MNP* cells) did not map as well and seemed to overfit specific voxels, suggesting that the resulting patterns are not biologically sound. These may reflect lower conservation of marker genes in immune cells, which can be addressed in the future by using scRNA-seq data from both species to optimize marker selection.

References

1. Human M1 10x. https://portal.brain-map.org/atlases-and-data/rnaseq/human-m1-10x.

2. Hodge, R. D. *et al.* Conserved cell types with divergent features in human versus mouse cortex. *Nature* **573**, 61–68 (2019).

3. Stewart, B. J. *et al.* Spatiotemporal immune zonation of the human kidney. *Science* **365**, 1461–1466 (2019).

4. 10X Genomics, Mouse Kidney Dataset. https://support.10xgenomics.com/spatial-gene-expression/datasets/1.1.0/V1_Mouse_Kidney.
